# Environmental and Biological Influences on Carbonate Precipitation Within Hot Spring Microbial Mats in Little Hot Creek, CA

**DOI:** 10.3389/fmicb.2018.01464

**Published:** 2018-07-13

**Authors:** Dylan T. Wilmeth, Hope A. Johnson, Blake W. Stamps, William M. Berelson, Bradley S. Stevenson, Heather S. Nunn, Sharon L. Grim, Megan L. Dillon, Olivia Paradis, Frank A. Corsetti, John R. Spear

**Affiliations:** ^1^Department of Earth Sciences, University of Southern California, Los Angeles, CA, United States; ^2^Department of Biological Science, California State University, Fullerton, Fullerton, CA, United States; ^3^Geo- Environmental- Microbiology Laboratory, Department of Civil and Environmental Engineering, Colorado School of Mines, Golden, CO, United States; ^4^Department of Microbiology and Plant Biology, University of Oklahoma, Norman, OK, United States; ^5^Geomicrobiology Laboratory, Department of Earth and Environmental Sciences, University of Michigan, Ann Arbor, MI, United States; ^6^Department of Earth and Planetary Sciences, University of California, Davis, Davis, CA, United States

**Keywords:** microbial mat, carbonate precipitation, biomineralization, carbon fixation, hot spring

## Abstract

Microbial mats are found in a variety of modern environments, with evidence for their presence as old as the Archean. There is much debate about the rates and conditions of processes that eventually lithify and preserve mats as microbialites. Here, we apply novel tracer experiments to quantify both mat biomass addition and the formation of CaCO_3_. Microbial mats from Little Hot Creek (LHC), California, contain calcium carbonate that formed within multiple mat layers, and thus constitute a good test case to investigate the relationship between the rate of microbial mat growth and carbonate precipitation. The laminated LHC mats were divided into four layers via color and fabric, and waters within and above the mat were collected to determine their carbonate saturation states. Samples of the microbial mat were also collected for 16S rRNA analysis of microbial communities in each layer. Rates of carbonate precipitation and carbon fixation were measured in the laboratory by incubating homogenized samples from each mat layer with δ^13^C-labeled HCO_3_^-^ for 24 h. Comparing these rates with those from experimental controls, poisoned with NaN_3_ and HgCl_2_, allowed for differences in biogenic and abiogenic precipitation to be determined. Carbon fixation rates were highest in the top layer of the mat (0.17% new organic carbon/day), which also contained the most phototrophs. Isotope-labeled carbonate was precipitated in all four layers of living and poisoned mat samples. In the top layer, the precipitation rate in living mat samples was negligible although abiotic precipitation occurred. In contrast, the bottom three layers exhibited biologically enhanced carbonate precipitation. The lack of correlation between rates of carbon fixation and biogenic carbonate precipitation suggests that processes other than autotrophy may play more significant roles in the preservation of mats as microbialites.

## Introduction

Microbial mats have been preserved within the rock record over 3.5 billion years (Walter et al., 1980; Noffke et al., 2006; Schopf, 2006; Bosak et al., 2009). Structures attributed to microbial precipitation of carbonate minerals, commonly termed microbialites, are particularly common in the geologic past (Awramik and Sprinkle, 1999; Grotzinger and Knoll, 1999; Riding, 2000; Bosak et al., 2013). Despite the ubiquity of microbial mats in a variety of modern aqueous environments, few appear to be mineralized in a way that would lead to the preservation of a microbialite. When identified, modern microbial mats that precipitate minerals provide potential analogs for the formation of ancient microbialites (Reid et al., 2000; Visscher et al., 2000; Dupraz et al., 2004, 2009; Dupraz and Visscher, 2005; Vasconcelos et al., 2006; Mata et al., 2012).

The metabolic activity within microbial mats and the physicochemical changes in the surrounding environment can influence mineral saturation states and induce mineral precipitation (Grotzinger and Knoll, 1999). This study defines mineralization that occurs when the activities of living microbes act to enhance precipitation as *biogenic precipitation* (see also “organomineralization *sensu stricto*” in Trichet and Defarge, 1995, “microbially induced precipitation” in Dupraz et al., 2009). Mineralization within mats that is independent of living microbial activity is defined as *abiogenic precipitation* (see also “biologically influenced precipitation” in Frankel and Bazylinski, 2003; Dupraz et al., 2009). Three factors can influence the precipitation of calcium carbonate within microbial mats, whether biogenic or abiogenic in nature: the concentrations of CO_3_^2-^ ions, Ca^2+^ ions, and surface chemistry or nucleation centers (Dupraz et al., 2009). The first two factors relate to the saturation state of calcium carbonate, defined as omega (Ω), the product of calcium and carbonate ion concentrations divided by the solubility constant *K*_sp_’ for the appropriate mineral (where the symbol ’ accounts for the activities of Ca^2+^ and CO_3_^2-^). When calcium carbonate is at equilibrium in solution, Ω = 1, with under- and over-saturated solutions bearing lower and higher values, respectively. The third factor relates to the potential for locations within microbial mats to serve as nuclei for carbonate minerals to form (Arp et al., 1999a; Dupraz et al., 2009).

Certain metabolisms have been studied as candidates for inducing biogenic carbonate precipitation, including oxygenic photosynthesis (Pentecost and Riding, 1986; Merz-Preiß and Riding, 1999; Riding, 2000), anoxygenic photosynthesis (Bundeleva et al., 2012), sulfate reduction (Visscher et al., 2000; Dupraz et al., 2004; Baumgartner et al., 2006; Gallagher et al., 2012), and anaerobic oxidation of methane (Michaelis et al., 2002). For example, cyanobacteria increase saturation through carbon fixation by raising pH and removing CO_2_. Cyanobacteria can also produce abundant exopolymeric substances which can serve as nucleation centers for carbonate precipitation (De Philippis et al., 1998; Obst et al., 2009). However, precipitation induced via cyanobacterial photosynthesis occurs primarily in freshwater environments with low dissolved inorganic carbon and high calcium concentrations, and is not correlated with areas of increased carbon fixation (Arp et al., 1999a,b, 2001; Merz-Preiß and Riding, 1999). In lakes with high dissolved inorganic carbon, aragonite precipitation is only associated with cyanobacteria which contain an external fibrous layer on cell walls (Couradeau et al., 2013; Gérard et al., 2013). Finally, internal accumulation of calcium has been observed in multiple cyanobacterial lineages from lacustrine environments and hot springs (Ragon et al., 2014; Cam et al., 2018). Therefore, external environmental factors, cellular morphologies, and intracellular chemistry can affect precipitation in microbial mats even when metabolisms that increase carbonate saturation are abundant.

One method used to examine biologically induced precipitation is the comparison of metabolic rates with patterns of carbonate mineralization within microbial mats. However, studies directly comparing rates of precipitation and metabolic activity remain limited. Within modern Bahamian stromatolites, laminae with high sulfate reduction rates coincide with zones of carbonate precipitation (Visscher et al., 2000; Dupraz et al., 2004). Rates of sulfate reduction were measured using incubations with ^35^S-labeled SO_4_^2-^, while carbonate precipitation was assessed through petrographic analysis (Visscher et al., 2000). In contrast, numerical models of metabolic impact on carbonate saturation hypothesize that sulfate reduction coupled with H_2_S oxidation decreases Ω (Aloisi, 2008). Bundeleva et al. (2012) found a correlation between carbonate precipitation and biomass production through anoxygenic photoheterotrophic growth with pure cultures of the proteobacterium *Rhodovulum* in supersaturated solutions (Ω = 10–120).

Measuring carbonate precipitation rates within microbial mats complements constraints on microbialite growth rates determined in studies of stromatolite accretion. Previous hypotheses have suggested that stromatolite laminae form daily via the diurnal activity of cyanobacteria (Doemel and Brock, 1974; Golubic and Focke, 1978; Pepe-Ranney et al., 2012). In contrast, studies of Holocene stromatolites using ^14^C dating suggest seasonal or even multiyear lamination rates (Chivas et al., 1990; Berelson et al., 2011; Petryshyn et al., 2012). The variability in stromatolite accretion rates is likely due to the variety of biogenic and abiogenic precipitation mechanisms within microbial mats. Comparing precipitation rates in relation to specific metabolisms or physicochemical processes in turn provides constraints for modern and ancient microbialite growth.

This study examines the effects of microbial metabolism and environmental factors on nascent carbonate precipitation within microbial mats from Little Hot Creek (LHC), California by focusing on the rate of carbon fixation into biomass and concomitant carbonate precipitation. Isotope labeling experiments were used to compare rates of carbonate precipitation and autotrophic production of organic carbon during incubations with ^13^C enriched bicarbonate. Rates of precipitation were compared between active and poisoned microbial mats to discern biogenic from abiogenic carbonate production.

## Site Description and Previous Work

**FIGURE 1:**
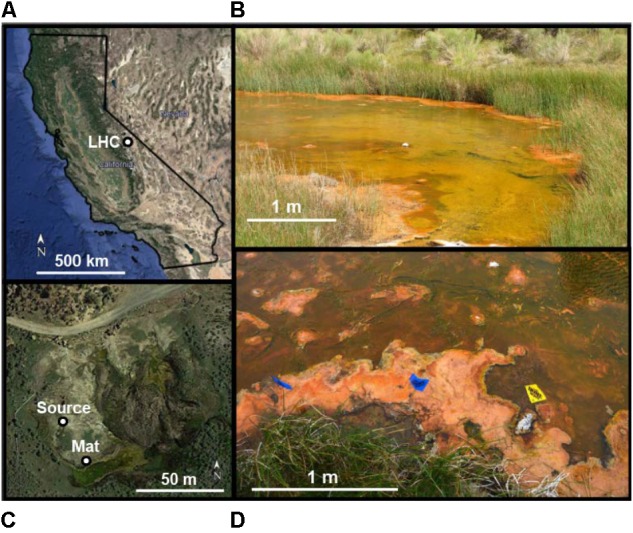
Location of Little Hot Creek (LHC) microbial mats. **(A)** Map of California. **(B)** View of mat location area looking east (downstream). Mat sampling and field geochemistry was performed on left-hand creek margin. Green areas toward the center of creek flow represent subaqueous mats, orange areas on creek margins represent mats at creek surfaces. **(C)** Aerial overview of LHC source and mat location. **(D)** Location of mat sampling and field geochemistry. Creek flow is from right to left. The yellow flag represents the Upstream location for field geochemistry, while the left blue flag represents the Downstream location (see **Table 1**). The orange surficial mat was sampled between the middle blue flag and the yellow flag (see **Figure 2A**).

Little Hot Creek is a stream sourced from hydrothermal springs within the Long Valley Caldera of eastern California (**Figures 1A,B**). The source of LHC is on the eastern flank of a resurgent dome formed by a rising magma chamber beneath the caldera (Sorey et al., 2003). Thermal waters emerge at ∼80°C from several vents (Vick et al., 2010). The vents discharge into LHC, where the waters cool to ∼50°C over the course of approximately 30 m. Several recent studies have characterized the microbiology and geochemistry of parts of the site. Vick et al. (2010) examined the microbial complement of the LHC source vents, and determined that groups related to the *Aquificae* and *Thermodesulfobacteria*, among others, were present in the hottest portions of the system, where the thermal waters discharge onto the surface. Source vent environments had circumneutral pH (6.75) and calcium concentrations ∼0.55 mM (Vick et al., 2010). Breitbart et al. (2004) examined phage–microbial interactions in LHC streams from several vent sources. While specific taxa were not described, the microbial communities at one location several meters downstream (74°C, pH 7.7) had a turnover rate of under one day based on labeled thymidine incubations (Breitbart et al., 2004). Dendrolitic cone structures in microbial mats from a cooler, adjacent pool (45°C) have also been studied as a potential analog for ancient microbialites (Bradley et al., 2017; Kraus et al., 2018).

Extensive microbial mats occur 25 m downstream from source waters, where flow velocity decreases as the creek bed widens from <1 to 8 m across (**Figure 1C**). Textures and mat thickness differ between wholly subaqueous mats and those at the creek surface. Subaqueous mats occur toward the center of the creek and are predominantly green with small orange patches (**Figure 1C**). Portions of the subaqueous mats several centimeters wide are partially detached from the creek bed, forming “rollover structures” (Hagadorn and Mcdowell, 2012) and exposing patches of underlying sediment. The resulting voids indicate a maximum mat thickness of 1–2 cm. In contrast, surficial mats are orange to tan, and usually extend from creek margins up to 1 m into LHC (**Figures 1C,D**), with smaller patches in the middle of the stream. Surficial mats rise up to 10 cm above the LHC creek bed, often reaching the creek’s surface. Surficial mat textures are clotted, and do not exhibit rollover features seen in subaqueous mats.

## Methods

### Field Geochemical Analysis

Microbial mats and creek waters were sampled ∼30 m downstream from LHC headwaters (**Figure 1B**). Temperature, pH, calcium concentrations, TCO_2_ (H_2_CO_3_ + HCO_3_^-^ + CO_3_^2-^), and δ^13^C of the TCO_2_ were measured in pore waters 1 and 5 cm deep within the sampled mat. Measurements were also made in stream waters 20 cm upstream, and 80 cm downstream from the mat. All field geochemical measurements were performed at the same time (June 2015), except for pH values within mat pore water, which were measured in August 2015. Temperature, TCO_2_, δ^13^C, calcium concentrations, and stream water pH values were virtually identical in June and August, increasing the confidence in using August pore water pH values to calculate saturation states. Saturation states of calcium carbonate within and around the LHC mat were calculated in CO2SYS (Pierrot et al., 2006) using pH data from a SevenGo Duo pH meter (Mettler Toledo) and TCO_2_ data obtained using a cavity ring-down spectrometer (CRDS; Picarro). Samples for calcium analysis were collected from the stream using a clean 20 mL syringe, filtered onsite with a 0.45 micron filter, and preserved with two drops of HCl. Calcium concentration was measured on the acidified sample using a microwave induced plasma-optical emission spectrometer (Agilent).

### Mat Extraction and Description

**FIGURE 2:**
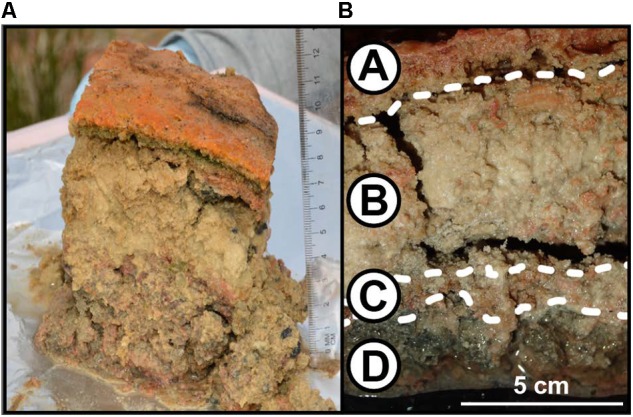
Structure of LHC microbial mats. **(A)** Microbial mat sample immediately after extraction. Note the distinct textural difference between the mat surface (Layer A) and the layers below (Layers B, C, and D). Scale bar is in cm. **(B)** Microbial mat after transport from field to laboratory. Layer A represents the mat surface, Layer D represents the mat bottom.

A piece of surficial microbial mat was selected for removal from LHC (**Figure 2A**). After extraction, the sampled mat was placed in a container lined with sterile aluminum foil, along with LHC water from around the mat to prevent desiccation, and was stored and transported on ice before laboratory analyses. The sampled LHC mat was divided into four layers based on texture and color (**Figure 2B**). Minerals from all layers were examined using an environmental scanning electron microscope (SEM). No preparation of sample was required for SEM examination.

### Incubation Experiments

The rates of biogenic carbonate precipitation and autotrophic production of organic carbon in each layer were determined using incubation experiments with ^13^C-labeled HCO_3_^-^. Prior to incubation, each layer was homogenized and separated into two sample groups – one for organic δ^13^C analysis and the other for inorganic δ^13^C analysis. In both groups, three sets of triplicate samples ∼2–3 mg each, (9 total) were prepared from each layer: (i) “Negative Control”: δ^13^C values of mat carbonates and organic carbon were obtained without incubation or addition of δ^13^C label; (ii) “Poisoned Control”: Mat samples were placed into 2 mL glass vials containing 10 μL of 300 mM NaN_3_ and saturated HgCl_2_ solution to poison the mat, filled and capped without headspace with LHC water amended with 5 mM HCO_3_^-^ and a δ^13^C label of +2000 ‰; (iii) “Living”: Mat samples were placed into 2 mL vials and filled and capped without headspace with LHC water amended with 5 mM HCO_3_^-^, and a δ^13^C label of +2000 ‰. **Table 1** gives the initial carbonate parameters for the stream and microbial mat pore water.

**Table 1:** Field measurements of stream water and pore water at LHC taken in June 2015.

Location	Upstream 20 cm	Downstream 80 cm	Mat porewater: 1 cm (A/B boundary)	Mat porewater: 5 cm (Layer B)
*T* (°C)	52.4	38.1	38.8	48.4
TCO_2_ (mM)	11.91	11.88	11.52	11.59
δ^13^C (‰)	–2.58	–2.49	–2.06	–2.23
pH	8.34	8.29	8.10^∗^	8.30^∗^
Ω	4.62	3.50	3.23	4.27

*^∗^Indicates measurements taken in August 2015. Upstream and Downstream locations are relative to the sampled mat.*

For both carbonate precipitation and organic carbon production experiments, Poisoned Controls and Living samples were incubated for 24 h at 40°C. To account for changing metabolism over a diel cycle, the incubator was set on a 12-h light/dark cycle. After incubation, samples for organic δ^13^C analyses were treated with 1M HCl until effervescence ceased. All incubations were washed with phosphate-buffered saline (PBS) three times by centrifugation, decanting of the supernatant, and suspension of the pellet, in order to remove labeled HCO_3_^-^ that was not incorporated into carbonates or organic material. After HCl treatment and the PBS wash, all samples were oven-dried at 60°C.

Dried samples were ground, weighed (1–3 mg) and measured for % C and δ^13^C on a Picarro CRDS (Santa Clara, CA, United States) (Supplementary Tables S1 and S2). Samples analyzed for carbonates were acidified with phosphoric acid in a closed vessel, with evolved CO_2_ passing into the Picarro CRDS. Organic carbon was analyzed on de-carbonated samples by oxidizing dried material at 1000°C in a Costech Elemental Analyzer and passing CO_2_ into the Picarro. The averages of triplicate δ^13^C measurements for Living and Poisoned Control incubations were compared with averages of Negative Control samples from respective layers. Heavier δ^13^C values in Living and Poisoned Control incubated samples compared to Negative Control samples indicated incorporation of labeled HCO_3_^-^ by carbonate precipitation (in the case of carbonate analysis) or production of autotrophic organic carbon (in samples combusted via EA).

An analysis of the significance of different isotope values for different treatments was determined by comparing δ^13^C triplicate averages (Δ_avg_) with distributions of potential Δ_avg_ values using bootstrap analyses (Efron, 1979; Efron and Tibshirani, 1986). After calculating the experimental Δ_avg_ between two triplicate averages ($\Delta_{\mathrm{avg}}^{exp}$), δ^13^C values from all six samples were resampled at random to create two novel triplicate sets. The difference in these two resampled triplicate sets produced a new Δ value ($\Delta_{\mathrm{avg}}^{res}$). δ^13^C values were resampled with replacement, which indicates that a single δ^13^C value can be selected multiple times. For example, resampling with replacement can produce two triplicates where all six δ^13^C values are the same, producing a $\Delta_{\mathrm{avg}}^{res}$ of 0. Repeating the resampling process 1000 times produced distributions of $\Delta_{\mathrm{avg}}^{res}$ values which were then compared with the measured $\Delta_{\mathrm{avg}}^{exp}$. Values of $\Delta_{\mathrm{avg}}^{exp}$ greater than 95% of $\Delta_{\mathrm{avg}}^{res}$ distributions (*p* = 0.05) were evaluated as non-random representations of δ^13^C that increased during incubation experiments, supporting the hypothesis that carbonate precipitation or carbon fixation occurred. Values of $\Delta_{\mathrm{avg}}^{exp}$ were also compared with the Δ_avg_ that could be produced by machine variability alone on two identical triplicates ($\Delta_{\mathrm{avg}}^{mac}$). The standard deviation (σ) for multiple CRDS δ^13^C measurements is conservatively ±0.1 ‰ (Subhas et al., 2015) σ for a triplicate average is 0.058 ‰ (0.1 × 3^-0.5^), and σ for Δ_avg_ is 0.082 ‰ [(0.058^2^ + 0.058^2^)^0.5^]. The $\Delta_{\mathrm{avg}}^{exp}$ values which were larger than 2σ (0.164 ‰) have less than 5% probability of machine variability as an origin, while $\Delta_{\mathrm{avg}}^{exp}$ values less than σ have a 14% or higher probability.

The amount of new inorganic or organic carbon produced during incubations was calculated using an isotope mass balance approach. Our primary assumption was that new carbonate or autotrophic organic carbon would incorporate the heavier δ^13^C value of the HCO_3_^-^ label with no significant fractionation. In making our isotope mass balance, we use the fractional abundance of ^13^C/(^12^C + ^13^C)] in Living and Poisoned Control samples with the bicarbonate label before and after incubation as per Hayes (1983).

(1) $$\begin{array}{c} Percent new growth=(1-(F_{\mathrm{Living}}-F_{{\mathrm{HCO}}_{3}^{-}})/(F_{Poisoned control}-F_{{\mathrm{HCO}}_{3}^{-}}))\times 100 \end{array}$$

*F* represents the ^13^C/^12^C ratio in each component measured, and HCO_3_^-^ denotes the bicarbonate spike value. Abiogenic carbonate production rates were calculated by replacing the fractional abundances for Living and Poisoned Control with Poisoned Control and Negative Control, respectively. The solution to Eq. 1 was turned into a rate by dividing percent new carbon production by the incubation time (24 h), and were expressed as % new carbon/day.

### DNA and Community Composition Analysis

The bacterial and archaeal composition of LHC mats was determined by sequencing amplified libraries of small subunit ribosomal RNA (16S rRNA) genes. Field samples of all layers were amplified and sequenced in triplicate, with subsequent single analyses of mat samples taken from each layer immediately before and after incubation.

Extraction of DNA from each sample was performed using the *Xpedition* Soil/Fecal DNA MiniPrep kit according to manufacturer’s instructions (Zymo Research Corp., Irvine, CA, United States). Extracted DNA was amplified using primers that spanned the V4 region of the 16S rRNA gene between positions 519 and 802 (*Escherichia coli* numbering), producing a product of approximately 266 bp. The primer pair amplified a broad distribution of both the Bacteria and Archaea (Klindworth et al., 2013). The forward primer (M13L-519F: 5′-**GTA AAA CGA CGG CCA GCA** CMG CCG CGG TAA-3′) contained the M13 forward primer (in bold), followed by the 16S rRNA gene-specific sequence (underlined) to allow for barcoding of each sample in a separate reaction (Stamps et al., 2016). The reverse primer (785R: 5′-TAC NVG GGT ATC TAA TCC-3′) was taken directly from the “S-D-Bact07850b-A-18” reverse primer in Klindworth et al. (2013).

Each 50 μL PCR reaction consisted of: 1× 5 PRIME HOT master mix (5 PRIME Inc., Gaithersburg, MD, United States), 0.2 μM of each primer, molecular grade water, and 4 μL of extracted template DNA. The thermal cycling used for PCR was the same as described in Stamps et al. (2016). Positive (*E. coli*) and negative (no template) controls were also amplified along with sample template reactions. The amplified DNA molecules were then purified using AMPure XP paramagnetic beads (Beckman Coulter Inc., Indianapolis, IN, United States) at a final concentration of 0.8× v/v. A second, six cycle PCR was used to add a unique 12 bp barcode to each previously amplified sample using a forward primer containing the unique barcode + M13 forward sequence (5′-3′) and the original 785R primer (A mapping file is available at 10.5281/zenodo.1067761). The final barcoded PCR products were again cleaned and concentrated using AMPure XP paramagnetic beads at a final concentration of 0.8× (v/v), quantified using the QuBit dsDNA HS assay (Life Technologies, Carlsbad, CA, United States), pooled in equimolar amounts, and concentrated to a final volume of 80 μL using two Amicon Ultra-0.5 mL 30K Centrifugal Filters (EMD Millipore, Billerica, MA, United States).

The final pooled library was sequenced using the Illumina MiSeq platform (Illumina, San Diego, CA, United States) and the PE250 V2 chemistry. After sequencing, reads were merged and de-multiplexed using QIIME (Caporaso et al., 2010), filtered at a minimum quality score of 20 before being clustered into sub-operational taxonomic units (sOTUs) using Deblur (Amir et al., 2017). Raw reads were deposited into the NCBI sequencing read archive (SRA) under the accession number SRX2830741. An R markdown notebook and all required data to recreate the 16S rRNA gene analyses presented here are available at 10.5281/zenodo.1067761. Taxonomy was assigned using mothur (Schloss et al., 2009) against the SILVA database (Release 128) (Pruesse et al., 2007).

## Results

### Environmental Characterization

The LHC mat sampled for biomass and precipitation experiments was a surficial mat ∼30 m from source waters, extending 50 cm from the shoreline into creek flow (**Figure 1D**). The total mat thickness was 10 cm with a gradational contact between the deepest layer (D) and the underlying sediment. Unconsolidated mineral crystals were noted within the mat as granulated textures during extraction, but no lithified structures such as carbonate crusts or continuous nodules or layers were observed. Temperature, pH, TCO_2_, and δ^13^C data from upstream, downstream, and pore waters within the mat collected prior to extraction are shown in **Table 1**. The temperature of surface waters decreased from 52.4°C upstream to 38.1°C downstream of the sampled mat. The temperature at the mat surface was 34.1°C, increasing to 48.4°C at 3 cm mat depth. The pH of stream waters above the mat in June 2015 decreased from 8.34 upstream to 8.29 downstream. The pH within mats in August 2015 increased from 8.10 to 8.30 with depth. TCO_2_ of creek waters decreased downstream, while δ^13^C values of TCO_2_ became slightly heavier. With increasing mat depth, TCO_2_ increased and δ^13^C values of TCO_2_ decreased (see **Table 1**). Mat pore waters were supersaturated with respect to calcium carbonate, with Ω increasing from 3.23 at 0.5 cm depth to 4.27 at 10 cm.

### A Layered Mat With Extensive Carbonate Precipitation

**FIGURE 3:**
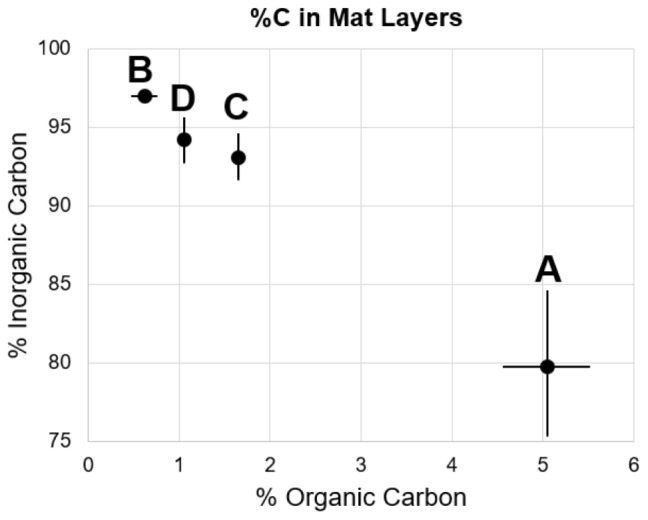
Percent organic and inorganic carbon of mat layers. Points represent triplicate averages, error bars represent one standard deviation.

The microbial mat contained four layers that were visually defined, labeled A through D from top to bottom (**Figure 2B**). Layer A was 1 cm thick and graded from bright orange at the mat–water interface to olive green at the boundary with Layer B. Layer A was extremely cohesive, with isolated pieces retaining shape when separated from the mat. Layer B was 3 cm thick, tan, and lacked the cohesiveness of Layer A. Layer C was 1 cm thick, light pink, and was more cohesive than Layers B or D, but less than Layer A. Layer D was 5 cm thick and gray, with similar consistency to Layer B. Weight percentages of organic and inorganic carbon were inversely correlated within these mat layers (**Figure 3** and Supplementary Table S2). Layer A had the highest amount of organic carbon (5.1 wt%) and the lowest amount of carbonate (79.7 wt%), while Layers B, C, and D varied between 0.6–1.7 wt% organic carbon and 93.1–96.9 wt% carbonate. Despite the high weight percentage carbonate, the mat was not lithified and was easily disarticulated with a spatula.

**FIGURE 4:**
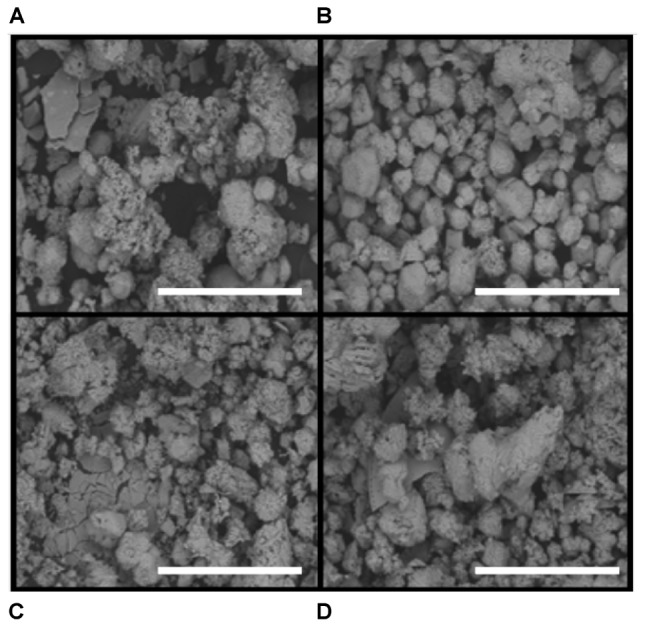
Scanning electron microscope (SEM) images of carbonate precipitates. Scale bar is 500 μm. **(A)** Layer A. **(B)** Layer B. **(C)** Layer C. **(D)** Layer D. Note the presence of euhedral crystals, particularly in Layer B, an indication of precipitation within mat layers as opposed to trapping and binding of detrital carbonates.

Upon removal of the organic matter, carbonate crystals were visible in all layers via SEM (**Figure 4**). Many carbonates formed individual rhombs up to 200 μm in diameter, with an average width of 50 μm. Carbonate crystals within LHC mats are euhedral and lack evidence for erosion such as rounding. Euhedral crystal morphologies indicate *in situ* precipitation, as opposed to trapping and binding of detrital carbonates. Crystal abundance broadly corresponded with measured carbonate weight percentage, with Layer A exhibiting less carbonate than Layers B through D (**Figure 3**). While the weight percentage of carbonate was high in all layers, the carbonate crystals were not interlinked and did not form a solid framework.

### Production Rates of Organic and Inorganic Carbon

**FIGURE 5:**
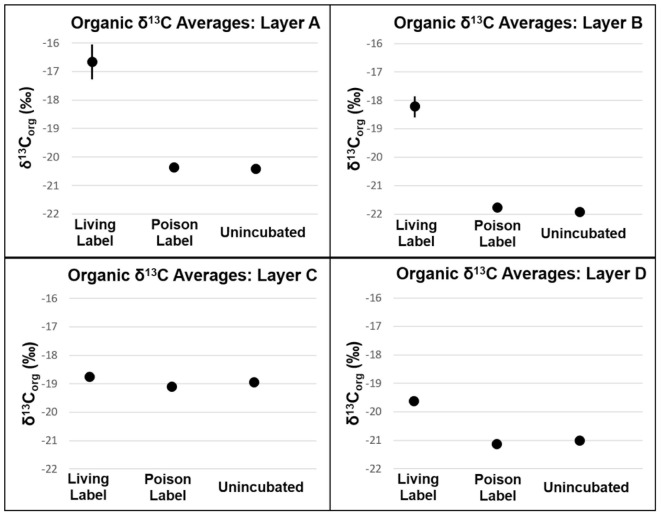
δ^13^C of organic carbon of mat layers after incubation experiments. Points represent triplicate averages, error bars represent one standard deviation.

**FIGURE 6:**
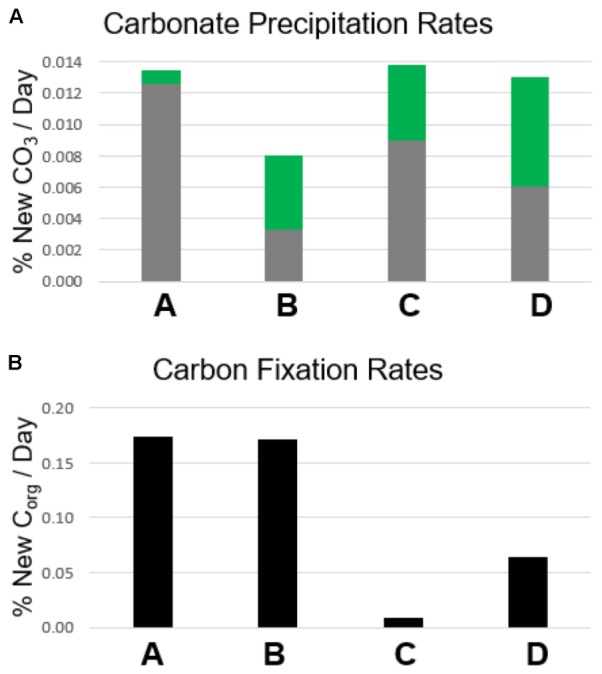
Rates of calcium carbonate precipitation and autotrophic organic carbon production. **(A)** Carbonate precipitation rates. Gray bars represent the amount of new carbonate precipitated in poisoned incubation samples as a percentage of the carbonate present prior to incubation. Green bars represent the additional amount of new carbonate precipitated in living incubation samples compared with poisoned incubation samples. **(B)** Carbon fixation rates. Bars represent the amount of organic carbon produced through carbon fixation in living incubation samples as a percentage of the organic carbon present prior to incubation.

Carbon fixation will produce more enriched δ^13^C_org_ values in mat samples incubated with labeled bicarbonate than un-incubated samples. In all layers, δ^13^C_org_ values were not heavier (*p* > 0.05, see Supplementary Table S3 and **Figure 5**) in poisoned than un-incubated samples, indicating little to no microbial activity in the poisoned incubations after addition of NaN_3_ and HgCl_2_ and also indicating that the addition of bicarbonate spike did not reside on the sample surface. In contrast, organic δ^13^C values were heavier in living than poisoned samples in each layer, implying uptake of labeled bicarbonate through autotrophic production of organic carbon (**Figure 5**). Layers A and B exhibited the highest rates of autotrophic carbon production (both 0.17% new organic carbon/day, *P* = 0.01, 0.003, respectively), followed by Layer D (0.07% new organic carbon/day, *P* = 0.01, see **Figure 6**). Production rates in Layer C were an order of magnitude lower than the other three layers (0.011% new organic carbon/day, *P* = 0.036).

**FIGURE 7:**
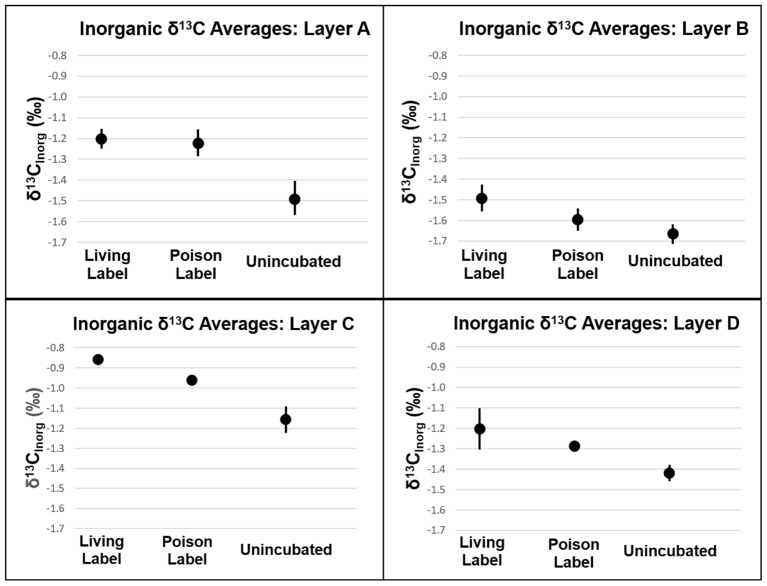
δ^13^C of inorganic carbon of mat layers after incubation experiments. Points represent triplicate averages (except for Layer B Living Label, which is the average of two samples), error bars represent one standard deviation.

Analogous to the carbon fixation experiment, carbonate precipitation will also produce heavier δ^13^C_inorg_ values in mat samples incubated with labeled bicarbonate than in un-incubated samples. In Layers A, C, and D, δ^13^C_inorg_ values were higher in poisoned than un-incubated samples (*P* = 0.021, 0.010, and 0.013, respectively, see **Figure 7** and Supplementary Table S3). The incorporation of labeled ^13^C into carbonates in poisoned samples provides evidence for abiogenic precipitation in the absence of active metabolisms. Poisoned samples from Layer B did not have heavier δ^13^C_inorg_ values than un-incubated samples (**Figure 6**, *P* = 0.071). This result shows that the incorporation of spike into carbonate is not an artifact of experiment design, but that abiotic precipitation did not occur in Layer B.

The lack of a difference between living and poisoned carbonate production in Layer A (**Figure 7**, *P* = 0.38) indicates that microbial activity/presence did not foster additional carbonate production at the mat’s surface. While the $\Delta_{\mathrm{avg}}^{exp}$ measured in Layer B was slightly lower than the 95% confidence interval in bootstrap analysis (*P* = 0.058), a comparison with $\Delta_{\mathrm{avg}}^{mac}$ shows only a 12% probability of machine variability explaining the observed data, similar to the probability of machine variability in Layer C (10%), which has $\Delta_{\mathrm{avg}}^{exp}$ higher than the 95% confidence interval (*P* = 0.034). The similarity in machine error probability between Layer C, which passes the bootstrap confidence interval test for precipitation, and Layer B, which falls just short of the confidence interval, increases confidence that some biogenic carbonate precipitation occurred in Layer B. Inorganic δ^13^C values in Layer D were heavier in living than in poisoned samples (*P* = 0.038). The incorporation of additional labeled ^13^C into carbonates in living relative to poisoned samples from Layers B, C, and D is evidence that microbial activity plays a role in increasing the carbonate content of these layers.

### Mat Communities With Depth

**FIGURE 8:**
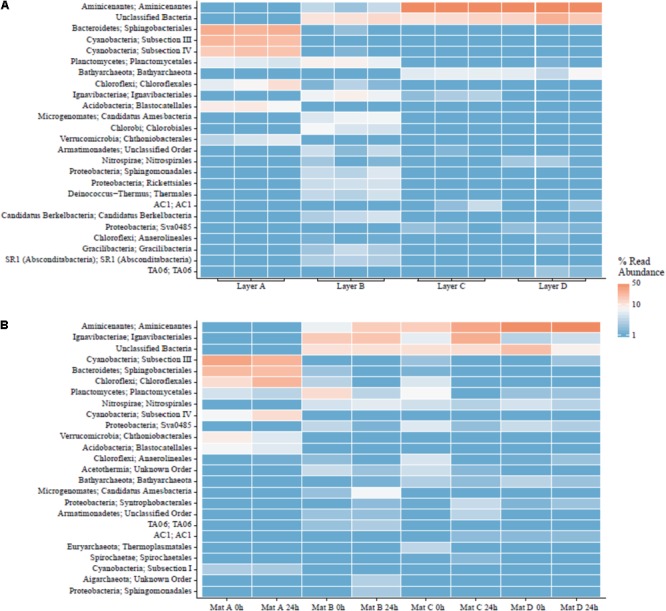
Major orders in LHC mat layers. **(A)** Field triplicates. **(B)** Laboratory samples prior to and after incubation experiments.

Cyanobacteria and Bacteroidetes were the most abundant phyla in the surface Layer A (see **Figure 8**). Members of the phylum Cyanobacteria composed ∼37–40% of the community, including the genera *Calothrix, Leptolyngbya, Synechococcus*, and *Phormidium*. The phylum Bacteroidetes (∼18–23%) held the most abundant taxa in Layer A, an uncultured member of the Saprospiraceae (17–22%). The rest of the community was predominantly composed of members of the phyla Chloroflexi (∼6–23%), Acidobacteria (∼5–9%), Verrucomicrobia (3–9%), and Planctomycetes (∼3–6%). Compared to samples preserved immediately in the field, the Chloroflexi from Layer A seemed to respond favorably during the 24 h experimental incubation, more than doubling in relative abundance (from ∼9 to 18%). The microbial community in Layer B may have contained abundant novel taxa, as no taxonomic classification was given to 10–12% of these sequences, and while of quality sequence, they remain unclassified. Ignavibacteria responded favorably during transportation prior to incubation, more than doubling from ∼7 to 16%. Additionally, Aminicenantes (Candidate Phylum OP8) increased from 5.9 to 15.1% during incubations. The other most abundant classified taxa in Layer B were members of the phyla Proteobacteria (3–9%), Chlorobi (4–7%), Planctomycetes (3–12%), and Chloroflexi (1–4%).

Layers C and D had very similar communities based on 16S rRNA gene analysis. Aminicenantes was the most abundant phylum in Layers C and D, at 15–73% and 46–67%, respectively. The phylum did not respond favorably during transportation in Layer C, decreasing from ∼70% from samples taken immediately in the field to 14.9 and 30.1% immediately prior to and after laboratory incubation, respectively. Unclassified taxa also formed a major fraction of the community (∼10–13% in C and ∼8–25% in D). Although the Ignavibacteriae were a relatively minor fraction of the microbial community in samples from Layer C preserved in the field (∼2%), they increased in relative abundance over fivefold during incubations (5.5–27%). The rest of the sequences in Layers C and D were members of the phyla Chloroflexi, Nitrospirae, Planctomycetes, and Bathyarchaeota, present as 2–5% of the community.

## Discussion

### Correlating Carbon Fixation With Carbonate Precipitation Within Mat Layers

Autotrophic production of organic carbon occurred in all layers of non-poisoned mats during the incubation experiments (**Figure 6**). The rate of this carbon fixation in Layers A and B at the top of the mat (0.17% new organic carbon/day) was more than twice as fast as that within Layer D (0.07% new organic carbon/day), and more than an order of magnitude greater than within Layer C (0.01% new organic carbon/day). Rates of carbon fixation in Layers A and B are nearly identical to rates measured by Bradley et al. (2017) from *in situ* incubations of surficial cones from LHC mats (0.15–0.17% new organic carbon/day), suggesting that meaningful rates can be determined using homogenized samples for the upper layers of the mat. Future comparisons between mats with preserved structure and homogenized mats have the potential to determine the effects of laboratory manipulations on rates in lower, light-limited and potentially anaerobic layers.

Autotrophic microbial communities have different abundances in each LHC mat layer. Previous incubations of hot spring microbial mats provide evidence for autotrophy by members of the phyla Cyanobacteria and Chloroflexi (van der Meer et al., 2005). Within LHC, members of the phylum Cyanobacteria were far more abundant in Layer A (35–39%) than in Layer B (0.5–1.5%) (**Figure 8**). Members of the phylum Chloroflexi were less abundant than Cyanobacteria in Layer A (8–24%), but relatively more abundant in Layer B (3–8%) (**Figure 8**). Despite differing abundances of Cyanobacteria and Chloroflexi, carbon fixation rates were nearly equal in Layers A and B (0.17% new organic carbon/day). Conversely, while Layers A and B had similar rates of carbon fixation with different microbial communities, Layers C and D had similar communities but Layer D had higher rates of carbon fixation (**Figure 6**). Chloroflexi are relatively common in both layers, but decreased from 14.2 to 2.3% during incubation in Layer C, potentially decreasing net carbon fixation. Finally, it is possible that future examination and identification of the ∼14–26% of unclassified taxa in Layers B, C, and D will yield further information on carbon-fixing organisms within the LHC mats.

Biogenic carbonate precipitation within the LHC mat occurred during incubation of Layers B, C, and D (**Figure 7** and Supplementary Table S3). In contrast, the highest rates of carbon fixation were in Layers A and B, with rates similar to *in situ* incubations of LHC cones (Bradley et al., 2017). Yet, the different carbonate precipitation rates in Layers A and B (**Figure 7** and Supplementary Table S3) did not support a positive correlation between carbon fixation and biogenic carbonate precipitation rates. While *in situ* rates within the lower LHC mat layers are not available for comparison, biogenic carbonate precipitation and carbon fixation did occur in these layers, and there was also no apparent correlation between the two processes. There also appears to be no correlation between biogenic and abiogenic carbonate precipitation in LHC mats. Both styles produce carbonate in Layers C and D, while only biogenic precipitation is prevalent in Layer B. Layer A had the highest rate of abiogenic precipitation with no major biogenic influence. Finally, abiogenic carbonate precipitation has no clear correlation with carbonate saturation states within LHC mats. Calcium carbonate was supersaturated at all points above and within mats, with Ω increasing with depth, from 3.23 at 0.5 cm (Layer A) to 4.27 at 10 cm (Layer D). However, abiogenic precipitation rates were highest in Layer A at the top of the mat, and were marginal within Layer B immediately below, with intermediate values in Layers C and D. This is in contrast with previous experiments in abiotic systems, which observe a positive correlation between precipitation rate and saturation (Shiraki and Brantley, 1995).

Layer B had the highest weight percentage of carbonate (96.9%, **Figure 3** and Supplementary Table S2), but only biogenic precipitation occurred during incubation. Conversely, Layer A has the highest abiogenic precipitation rates, and total precipitation rates (biogenic + abiogenic) equivalent to those of Layers C and D, but the lowest carbonate percentage (79.7%). One explanation for this discrepancy is spatial differentiation of communities as LHC mats grow over time. Taxa with higher motility have the potential to move to more habitable environments when conditions become less habitable (Stal, 1995; Nadeau et al., 1999). Cyanobacteria have been shown to exhibit motile behavior in reaction to light availability, ultraviolet radiation, and chemical gradients within mats (Richardson and Castenholz, 1987; Bebout and Garcia-Pichel, 1995; Ramsing et al., 1997). Carbonate precipitation has the potential to limit light availability in LHC mats, forcing motile photoautotrophs to move upward, differentiating a thin, relatively organic-rich and carbonate-poor layer (Layer A) on top of carbonate-rich, organic-poor layers below (Layers B, C, and D).

### Precipitation Rates Compared With Microbialite Formation

Precipitation rates from LHC mats not only provide valuable information about the dynamics of modern mat growth, but can also help constrain conditions for microbialite formation and preservation in deep time. Averaging all four layers, LHC mats are 91 wt% carbonate, with a precipitation rate of 0.012% new carbonate per day. The average dry sample mass is 0.002 mg. Therefore, a typical incubation sample had 0.0018 g of carbonate (0.002 grams total × 0.91 grams carbonate/grams total), and 2.2 E-7 grams of carbonate are precipitated every day (0.0018 grams carbonate × 0.00012 grams new carbonate/grams carbonate). At measured rates, a lithified microbialite with 100 wt% carbonate would take two more years to produce. This hypothesis will be easily testable with future observations of LHC mats.

There are three potential solutions to the discrepancy between the rates of precipitation observed within LHC mats and the lack of lithified microbialites in the same location. (i) The incubation experiments represent a “snapshot” of microbial mat processes instead of long-term, steady state conditions. While the short-term nature of the experiments explains the differences between laboratory and field observations, the explanation does not include, perhaps, a specific process that inhibits microbialite formation at LHC. While a few clades changed in abundance during transportation or incubation, the majority remained relatively stable, indicating that major changes in mat community composition during experiments are unlikely to explain the discrepancy. (ii) Metabolisms within LHC mats in the field inhibit precipitation or promote dissolution which may not have been recognized in the lab experiments. Although the communities of each layer during incubations are relatively representative of field abundances, we introduced more light and aerobic conditions to lower layers such as C and D. Such changes could promote metabolisms that are not normally active in field communities, either processes that promote carbonate precipitation, or inhibit carbonate dissolution. (iii) Organic carbon production dilutes carbonate mineralization. In this scenario, carbonate precipitation in LHC mats could be equal or potentially higher than observed in the incubation experiments. However, if production rates of organic carbon are consistently higher than carbonate precipitation rates, then the microbial mat will never completely lithify into a microbialite (see Supplementary Materials). While all three explanations merit further investigation on mats from LHC and other environments, dilution of carbonate mineralization through organic carbon production provides the best explanation with the current set of observations.

Hypotheses for ancient microbial growth rates vary between short-term diurnal cycles based on modern cyanobacterial motility (Doemel and Brock, 1974; Golubic and Focke, 1978), and slower growth over many months or years (Chivas et al., 1990; Petryshyn et al., 2012; Frantz et al., 2014). Evidence from LHC suggests that elements of both ideas are at work in modern mats. Patterns of LHC mat communities and carbonate growth suggest that cyanobacterial motility does separate modern mats into upper organic-rich and lower carbonate-rich zones, but on slower timescales than thought by previous hypotheses. At observed precipitation rates, a minimum of 23 years is required for a hypothetical LHC mat starting with no carbonate present to fully lithify, all things being equal. However, if production rates of organic carbon consistently outpace carbonate precipitation rates in this mat, it is possible that the mats may never become fully lithified. It is possible to speculate that the paucity of microbialites versus the ubiquity of microbial mats in modern environments may reside in the balance between rates of organic carbon production versus carbonate precipitation, where times of abundant microbialite formation in the past may represent conditions where carbonate precipitation rates were equal to or greater than microbial growth rates.

Our study of 1-day growth rate experiments shows (i) Rates of autotrophic carbon fixation are not correlated with rates of biogenic carbonate precipitation within certain microbial mats. The results corroborate previous work demonstrating that carbon fixation only precipitates calcium carbonate in specific chemical environments (Merz-Preiß and Riding, 1999; Arp et al., 2001). The uppermost layers of LHC mats have the highest carbon fixation rates as well as abundant autotrophic communities, but biogenic precipitation only occurred in Layers B, C, and D. (ii) Mats with consistently higher rates of organic carbon production than carbonate precipitation are unlikely to produce lithified microbialites. This hypothesis is supported by recent research indicating higher rates of primary productivity in unlithified microbial mats than within recently formed stromatolites (Schuler et al., 2017). (iii) Individual microbial mat layers can host both biogenic and abiogenic carbonate precipitation. Abiogenic precipitation occurred in the uppermost LHC layer, and in concert with biogenic precipitation in the lower two layers. (iv) Layers with the highest carbonate production rates do not always correspond with areas of high carbonate percentage. The uppermost LHC layer had the lowest percentage of carbonate (79.7%), but the highest rate of abiogenic carbonate precipitation, while the immediately adjacent layer was 96.9% carbonate, but had the lowest rates of total carbonate precipitation during incubations. Comparing differences in carbonate percentage and production rate can potentially provide growth histories of microbial mats.

## Author Contributions

DW assisted with mat collection, field geochemistry, laboratory experiments, data analysis and interpretation, and wrote the initial manuscript draft. SG and MD assisted with mat collection, field geochemistry, laboratory experiments, data analysis and interpretation. HJ and WB designed and ran laboratory analyses, and assisted with geochemical interpretation. BSS, BWS, and HN performed 16S rRNA analyses, and assisted with biological interpretation. OP assisted with field geochemical sampling and saturation state calculation. FC co-directed and secured funding for the 2015 International Geobiology Course, and assisted with interpreting significance to modern and ancient lithified microbialites. JS co-directed and secured funding for the 2015 International Geobiology Course. All authors contributed in the writing and editing of the manuscript.

## Conflict of Interest Statement

The authors declare that the research was conducted in the absence of any commercial or financial relationships that could be construed as a potential conflict of interest.
