# Supplementary material for: Environmental and Biological Influences on Carbonate Precipitation Within Hot Spring Microbial Mats in Little Hot Creek, CA
Source: Front Microbiol. 2018 Jul 13;9:1464. doi: 10.3389/fmicb.2018.01464 (PMC6053513; doi:10.3389/fmicb.2018.01464)
Supplement: Supplementary file 1 [file Table_1.DOCX]

Supplementary Table 1:

δ^13^C values for inorganic and organic carbon during incubation experiments. SD signifies one standard deviation for the triplicate average.

| **Layer** | **Org/Inorg δ^13^C** | **Living/Poisoned/Un-Incubated** | **Triplicate** | **δ^13^C** | **Avg. δ13C** | **SD** |
| --- | --- | --- | --- | --- | --- | --- |
| A | Inorg | Living Label | 1 | -1.14 | -1.2 | 0.05 |
| A | Inorg | Living Label | 2 | -1.22 |  |  |
| A | Inorg | Living Label | 3 | -1.25 |  |  |
|  |  |  |  |  |  |  |
| A | Inorg | Poisoned Label | 1 | -1.22 | -1.22 | 0.08 |
| A | Inorg | Poisoned Label | 2 | -1.13 |  |  |
| A | Inorg | Poisoned Label | 3 | -1.32 |  |  |
|  |  |  |  |  |  |  |
| A | Inorg | Un-Incubated | 1 | -1.43 | -1.49 | 0.06 |
| A | Inorg | Un-Incubated | 2 | -1.58 |  |  |
| A | Inorg | Un-Incubated | 3 | -1.47 |  |  |
|  |  |  |  |  |  |  |
| A | Org | Living Label | 1 | -15.87 | -16.67 | 0.85 |
| A | Org | Living Label | 2 | -17.85 |  |  |
| A | Org | Living Label | 3 | -16.28 |  |  |
|  |  |  |  |  |  |  |
| A | Org | Poisoned Label | 1 | -20.41 | -20.37 | 0.04 |
| A | Org | Poisoned Label | 2 | -20.32 |  |  |
| A | Org | Poisoned Label | 3 |  |  |  |
|  |  |  |  |  |  |  |
| A | Org | Un-Incubated | 1 | -20.32 | -20.42 | 0.12 |
| A | Org | Un-Incubated | 2 | -20.59 |  |  |
| A | Org | Un-Incubated | 3 | -20.36 |  |  |
|  |  |  |  |  |  |  |
|  |  |  |  |  |  |  |
| **Layer** | **Org/Inorg δ^13^C** | **Living/Poisoned/Un-Incubated** | **Triplicate** | **δ^13^C** | **Avg. δ13C** | **SD** |
| B | Inorg | Living Label | 1 |  | -1.5 | 0.06 |
| B | Inorg | Living Label | 2 | -1.55 |  |  |
| B | Inorg | Living Label | 3 | -1.44 |  |  |
|  |  |  |  |  |  |  |
| B | Inorg | Poisoned Label | 1 | -1.62 | -1.6 | 0.04 |
| B | Inorg | Poisoned Label | 2 | -1.54 |  |  |
| B | Inorg | Poisoned Label | 3 | -1.63 |  |  |
|  |  |  |  |  |  |  |
| B | Inorg | Un-Incubated | 1 | -1.61 | -1.67 | 0.05 |
| B | Inorg | Un-Incubated | 2 | -1.74 |  |  |
| B | Inorg | Un-Incubated | 3 | -1.65 |  |  |
|  |  |  |  |  |  |  |
| B | Org | Living Label | 1 | -18.35 | -18.22 | 0.26 |
| B | Org | Living Label | 2 | -18.45 |  |  |
| B | Org | Living Label | 3 | -17.86 |  |  |
|  |  |  |  |  |  |  |
| B | Org | Poisoned Label | 1 | -21.73 | -21.78 | 0.08 |
| B | Org | Poisoned Label | 2 | -21.73 |  |  |
| B | Org | Poisoned Label | 3 | -21.89 |  |  |
|  |  |  |  |  |  |  |
| B | Org | Un-Incubated | 1 | -21.85 | -21.93 | 0.12 |
| B | Org | Un-Incubated | 2 | -21.85 |  |  |
| B | Org | Un-Incubated | 3 | -22.1 |  |  |
|  |  |  |  |  |  |  |
|  |  |  |  |  |  |  |
|  |  |  |  |  |  |  |
|  |  |  |  |  |  |  |
| **Layer** | **Org/Inorg δ^13^C** | **Living/Poisoned/Un-Incubated** | **Triplicate** | **δ^13^C** | **Avg. δ13C** | **SD** |
| C | Inorg | Living Label | 1 | -0.84 | -0.86 | 0.02 |
| C | Inorg | Living Label | 2 | -0.85 |  |  |
| C | Inorg | Living Label | 3 | -0.89 |  |  |
|  |  |  |  |  |  |  |
| C | Inorg | Poisoned Label | 1 | -0.96 | -0.96 | 0.06 |
| C | Inorg | Poisoned Label | 2 | -1.04 |  |  |
| C | Inorg | Poisoned Label | 3 | -0.89 |  |  |
|  |  |  |  |  |  |  |
| C | Inorg | Un-Incubated | 1 | -1.14 | -1.16 | 0.02 |
| C | Inorg | Un-Incubated | 2 | -1.19 |  |  |
| C | Inorg | Un-Incubated | 3 | -1.14 |  |  |
|  |  |  |  |  |  |  |
| C | Org | Living Label | 1 | -18.9 | -18.77 | 0.18 |
| C | Org | Living Label | 2 | -18.89 |  |  |
| C | Org | Living Label | 3 | -18.51 |  |  |
|  |  |  |  |  |  |  |
| C | Org | Poisoned Label | 1 | -18.99 | -19.11 | 0.12 |
| C | Org | Poisoned Label | 2 | -19.07 |  |  |
| C | Org | Poisoned Label | 3 | -19.28 |  |  |
|  |  |  |  |  |  |  |
| C | Org | Un-Incubated | 1 | -18.97 | -18.97 | 0.13 |
| C | Org | Un-Incubated | 2 | -18.8 |  |  |
| C | Org | Un-Incubated | 3 | -19.13 |  |  |
|  |  |  |  |  |  |  |
|  |  |  |  |  |  |  |
|  |  |  |  |  |  |  |
|  |  |  |  |  |  |  |
| **Layer** | **Org/Inorg δ^13^C** | **Living/Poisoned/Un-Incubated** | **Triplicate** | **δ^13^C** | **Avg. δ13C** | **SD** |
| D | Inorg | Living Label | 1 | -1.16 | -1.21 | 0.1 |
| D | Inorg | Living Label | 2 | -1.25 |  |  |
| D | Inorg | Living Label | 3 | -1.01 |  |  |
|  |  |  |  |  |  |  |
| D | Inorg | Poisoned Label | 1 | -1.26 | -1.29 | 0.02 |
| D | Inorg | Poisoned Label | 2 | -1.29 |  |  |
| D | Inorg | Poisoned Label | 3 | -1.32 |  |  |
|  |  |  |  |  |  |  |
| D | Inorg | Un-Incubated | 1 | -1.44 | -1.42 | 0.04 |
| D | Inorg | Un-Incubated | 2 | -1.37 |  |  |
| D | Inorg | Un-Incubated | 3 | -1.45 |  |  |
|  |  |  |  |  |  |  |
| D | Org | Living Label | 1 | -19.4 | -19.63 | 0.16 |
| D | Org | Living Label | 2 | -19.74 |  |  |
| D | Org | Living Label | 3 | -19.75 |  |  |
|  |  |  |  |  |  |  |
| D | Org | Poisoned Label | 1 | -20.87 | -21.15 | 0.34 |
| D | Org | Poisoned Label | 2 | -20.95 |  |  |
| D | Org | Poisoned Label | 3 | -21.63 |  |  |
|  |  |  |  |  |  |  |
| D | Org | Un-Incubated | 1 | -20.88 | -21.03 | 0.1 |
| D | Org | Un-Incubated | 2 | -21.11 |  |  |
| D | Org | Un-Incubated | 3 | -21.09 |  |  |
