# Supplementary material for: Environmental and Biological Influences on Carbonate Precipitation Within Hot Spring Microbial Mats in Little Hot Creek, CA
Source: Front Microbiol. 2018 Jul 13;9:1464. doi: 10.3389/fmicb.2018.01464 (PMC6053513; doi:10.3389/fmicb.2018.01464)
Supplement: Supplementary file 2 [file Table_2.DOCX]

Supplementary Table 2:

Percent organic and inorganic carbon in all mat layers. SD signifies one standard deviation for the triplicate average.

| **Layer** | **Organic Replicate** | **wt% C_org_** | **Average wt% C_org_** | **SD** |  | **Inorganic Replicate** | **wt%CaCO_3_** | **Average wt%CaCO_3_** | **SD** |
| --- | --- | --- | --- | --- | --- | --- | --- | --- | --- |
| **A** | 1 | 4.8 | 5.1 | 0.43 |  | 1 | 84.2 | 79.7 | 7.48 |
| **A** | 2 | 5.7 |  |  |  | 2 | 69.2 |  |  |
| **A** | 3 | 4.7 |  |  |  | 3 | 85.8 |  |  |
|  |  |  |  |  |  |  |  |  |  |
| **B** | 1 | 0.7 | 0.6 | 0.12 |  | 1 | 96.7 | 96.9 | 0.39 |
| **B** | 2 | 0.7 |  |  |  | 2 | 97.5 |  |  |
| **B** | 3 | 0.5 |  |  |  | 3 | 96.7 |  |  |
|  |  |  |  |  |  |  |  |  |  |
| **C** | 1 | 1.7 | 1.7 | 0.018 |  | 1 | 93.3 | 93.1 | 1.71 |
| **C** | 2 | 1.6 |  |  |  | 2 | 95.0 |  |  |
| **C** | 3 | 1.7 |  |  |  | 3 | 90.8 |  |  |
|  |  |  |  |  |  |  |  |  |  |
| **D** | 1 | 1.1 | 1.1 | 0.072 |  | 1 | 95.0 | 94.2 | 1.18 |
| **D** | 2 | 1.0 |  |  |  | 2 | 92.5 |  |  |
| **D** | 3 | 1.1 |  |  |  | 3 | 95.0 |  |  |
