# Supplementary material for: Environmental and Biological Influences on Carbonate Precipitation Within Hot Spring Microbial Mats in Little Hot Creek, CA
Source: Front Microbiol. 2018 Jul 13;9:1464. doi: 10.3389/fmicb.2018.01464 (PMC6053513; doi:10.3389/fmicb.2018.01464)
Supplement: Supplementary file 3 [file Table_3.DOCX]

Supplementary Table 3:

Differences in organic and inorganic carbon triplicate averages (Δ_avg_**).** L = Living Label, P = Poisoned Label, N = Unincubated. Δ_avg_ values with bootstrap p-values < 0.05 and probability of machine variability below 5% represent carbon fixation and carbonate precipitation in organic and inorganic carbon samples, respectively. Exceptions include: *) In Layer B, the Δ_avg_ was calculated between 3 Living Label and 2 Poisoned Label samples, instead of two triplicates like every other comparison. The Δ_avg_, bootstrap p-value, and probability of machine variability are close enough to Layer C, which meets the bootstrap and machine variability tests, to merit an interpretation of biogenic precipitation.

| **ORGANIC CARBON** |  | | | | | | | |
| --- | --- | --- | --- | --- | --- | --- | --- | --- |
| **Layer** | **A** | | **B** | | **C** | | **D** | |
| **Sample Groups Compared** | P - N | L - P | P - N | L - P | P - N | L - P | P - N | L - P |
| **Difference in Average δ^13^C (Δ_avg_)** | 0.058 | 3.70 | 0.15 | 3.56 | -0.15 | 0.35 | -0.12 | 1.5 |
| **% New Organic Carbon/Day** | 0.0027 | 0.17 | 0.0032 | 0.17 | -0.0023 | 0.012 | -0.0083 | 0.073 |
| **Bootstrap p-value (one-tailed T-test)** | 0.29 | 0.010 | 0.076 | 0.0030 | 0.12 | 0.036 | 0.28 | 0.010 |
| **Probability of machine variability** | 25% | <0.01% | 4% | <0.01% | 4% | <0.01% | 7% | <0.01% |
| **Autotrophy** |  | Present |  | Present |  | Present |  | Present |
|  | | | | | | | | |
| **INORGANIC CARBON** |  | | | | | | | |
| **Layer** | **A** | | **B** | | **C** | | **D** | |
| **Sample Groups Compared** | P - N | L - P | P - N | L - P | P - N | L - P | P - N | L - P |
| **Difference in Average δ^13^C (Δ_avg_)** | 0.23 | 0.02 | 0.07 | 0.10 | 0.19 | 0.10 | 0.13 | 0.15 |
| **% New Inorganic Carbon/Day** | 0.013 | 0.00093 | 0.0033 | 0.0047 | 0.0090 | 0.0048 | 0.0061 | 0.0070 |
| **Bootstrap p-value (one-tailed T-test)** | 0.021 | 0.38 | 0.071 | 0.058 | 0.010 | 0.034 | 0.013 | 0.038 |
| **Probability of machine variability** | 0.3% | 43% | 19% | 12% | 1% | 10% | 5% | 1% |
| **Carbonate Precipitation** | Abiogenic |  |  | Biogenic* | Abiogenic | Biogenic | Abiogenic | Biogenic |
