# Supplementary material for: Environmental and Biological Influences on Carbonate Precipitation Within Hot Spring Microbial Mats in Little Hot Creek, CA
Source: Front Microbiol. 2018 Jul 13;9:1464. doi: 10.3389/fmicb.2018.01464 (PMC6053513; doi:10.3389/fmicb.2018.01464)
Supplement: Supplementary file 4 [file Data_Sheet_1.DOCX]

SUPPLEMENTARY MATERIAL

*Production Rates of Carbonate and Organic Carbon by Volume*

Rates of carbonate precipitation and carbon fixation were calculated as % carbon/day. Respective volumes of carbonate and organic carbon produced per day can be estimated using weight percents, densities, and production rates of carbonate and organic matter. The average dry mass of samples was 0.002 g. Averaging all four layers, carbonate was 91 weight % of LHC mats. The composition of 0.002 g dry mat mass would therefore be 0.0018 g carbonate, and 0.0002 g organic carbon. At a precipitation rate of 0.012% new carbonate/day, 2.16 E-7 grams carbonate would be added daily. Carbon fixation rates of 0.1% new carbon/day would result in daily organic matter production of 2.08 E-5 grams. Dividing these rates by the densities of calcium carbonate (2.7 g/cm3) and organic matter (~1.5 g/cm3) results in 8 E-8 cm3 carbonate produced/day and 1.4 E-7 cm3 organic carbon produced/day. Therefore, carbon fixation produces ~1.7 times more mat volume on average than carbonate precipitation.
